# Edge-Based Color Image Segmentation Using Particle Motion in a Vector Image Field Derived from Local Color Distance Images

**DOI:** 10.3390/jimaging6070072

**Published:** 2020-07-16

**Authors:** Wutthichai Phornphatcharaphong, Nawapak Eua-Anant

**Affiliations:** Department of Computer Engineering, Faculty of Engineering, Khon Kaen University, Khon Kaen 40002, Thailand; wutthichai@kkumail.com

**Keywords:** color image segmentation, particle motion, local color distance images, normal compressive vector field, edge vector field

## Abstract

This paper presents an edge-based color image segmentation approach, derived from the method of particle motion in a vector image field, which could previously be applied only to monochrome images. Rather than using an edge vector field derived from a gradient vector field and a normal compressive vector field derived from a Laplacian-gradient vector field, two novel orthogonal vector fields were directly computed from a color image, one parallel and another orthogonal to the edges. These were then used in the model to force a particle to move along the object edges. The normal compressive vector field is created from the collection of the center-to-centroid vectors of local color distance images. The edge vector field is later derived from the normal compressive vector field so as to obtain a vector field analogous to a Hamiltonian gradient vector field. Using the PASCAL Visual Object Classes Challenge 2012 (VOC2012), the Berkeley Segmentation Data Set, and Benchmarks 500 (BSDS500), the benchmark score of the proposed method is provided in comparison to those of the traditional particle motion in a vector image field (PMVIF), Watershed, simple linear iterative clustering (SLIC), K-means, mean shift, and J-value segmentation (JSEG). The proposed method yields better Rand index (RI), global consistency error (GCE), normalized variation of information (NVI), boundary displacement error (BDE), Dice coefficients, faster computation time, and noise resistance.

## 1. Introduction

In digital image processing, image segmentation that reduces the amount of unnecessary data and preserves the important information needed for analysis plays an important role in image analysis. In general, image segmentation gathers pixels displaying similar characteristics within the same areas and converts them into regions. Among the various techniques, image segmentation methods can be divided into two main groups: machine learning image segmentation and classical image segmentation. First, machine learning image segmentation is a method by which a program can learn and segment an object by itself, without adjusting the program further. There are three types of machining approaches: supervised, unsupervised, and reinforcement learning methods. Supervised methods use a training dataset containing ground truth data to train artificial neural networks to map between input images and segmented results (see the survey [1]). However, the training process is computationally intensive and the ground truth construction that requires manual labeling by experts is labor-intensive. Additionally, when a new object class is added, the whole training dataset must be thoroughly reconstructed and the time-consuming training process must be repeated. In contrast, the unsupervised method does not require a dataset for training. Instead, the result of each iteration is recursively input to the program to adjust its parameters. This type of approach, such as K-means [2,3], mean shift [4,5], and JSEG [6,7], etc., is often more effective and more tolerant of unusual or unpredictable situations. However, the unsupervised methods are usually time-consuming due to its iterative processes embedded in the methods. Finally, the reinforcement learning method uses the reward and punishment techniques from environmental analysis for learning to drive the agent to the target. This method requires a large number of iterations for training of the agent to get a reward [8,9,10]. Second, classical image segmentation is a low-level image processing approach that tries to extract information without knowing the truth. Although, nowadays, machine learning image segmentation is state-of-the-art [11], classical image segmentation is still necessary in cases in which segmentation does not have ground truth images or there is a time constraint. Classical image segmentation also helps to create ground truth data in training datasets for machine learning techniques. Classical image segmentation techniques are comprised of thresholding-based, edge-based, region-based, and graph-based techniques. Thresholding-based techniques are divided into three types [12]: global thresholding, local thresholding, and adaptive thresholding. First, global thresholding weighs the distribution of intensity in the histogram to determine the threshold for separating objects from the background [13,14,15]. Second, local thresholding is used when a single threshold is not possible for images with uneven illumination or shadows. In such a case, it is necessary to use a sub-image to select the threshold [16]. Third, adaptive thresholding makes a calculation of the threshold by a window to find the intensity from the neighbor pixel [17,18]. The edge-based techniques, such as zero-crossing [19], Active Canny [20], PMVIF [21,22,23], EdgeFlow [24], and PointFlow [25], extract object boundaries in the image by creating contours around the objects. The region-based techniques, such as watershed [26,27], are based on the principle of grouping pixels with similar properties into the same regions or the same objects. Finally, the graph-based techniques, such as graph cuts [28,29], normalized cuts [30,31], Superpixel [32,33], and SLIC [34,35] proceed by grouping pixels according to graph theory. The methods mentioned above have various strengths and weaknesses, such as segmentation accuracy, processing time, flexibility, ease of use, and robustness to noise. For example, some algorithms can be applied only to grayscale images while some are only available in the RGB color space. Some methods are not suitable for real-time use or have to adjust too many parameters.

This paper introduces an edge-based classical image segmentation algorithm for a color image using particle motion in a vector image field derived from local color distance images (PMLCD). It is developed from the PMVIF algorithm that is known to have a fast computation time and yields closed boundaries but can be applied only to grayscale images. In the PMVIF algorithm, two vector fields, namely, the normal compressive vector field and the edge vector field, derived from derivatives of grayscale images, are used to force the particle to move along the object edges, which results in closed particle trajectories that resemble the object boundaries. In order to extend this principle to a color image segmentation task, the new formulae for computing the normal compressive vector field and the edge vector field, derived from the local color distance images, are introduced. The method proposed in this paper can be used not only with color images but also multichannel images such as hyperspectral images.

The rest of the paper is organized as follows: Section 2 describes the principle of the PMVIF algorithm; Section 3 describes the developed color image segmentation using particle motion in a vector image field derived from local color distance images; Section 4 presents the experimental validations and benchmarking of the proposed algorithm; finally, conclusions are drawn in Section 5.

## 2. Background to Particle Motion in a Vector Image Field

This section describes the principle of a traditional boundary extraction algorithm based on particle motion in a vector image field (PMVIF), which is an edge-based classical image segmentation approach. In general, in an N-dimensional space, a boundary can be explicitly represented by a manifold of dimension N-1 interfacing between regions of different attributes; for example, a close curve in a two-dimensional space. However, in a discretized image where a set of pixels or voxels is the only class that can exist, explicit representations of region boundaries, such as a curve or a surface, are difficult to encode. In this case, a normal compressive vector field [21,22,23], where all vectors are normal and point to the nearest interface, providing information about the direction to the nearest boundary, is more suitable to be used as an implicit boundary representation. Nevertheless, the normal compressive vector field itself only provides information about the location of the boundary but cannot offer any clues regarding the direction for tracking edges. In order to be able to locate and track a boundary simultaneously, another vector field containing vectors parallel to edges—namely an edge vector field—combined with the normal compressive vector field is required. The concept of using two such orthogonal vector fields for boundary extraction in a grayscale image was introduced in the PMVIF algorithm, where the gradient–Laplacian vector field used as a normal compressive vector field and the Hamiltonian gradient vector field used as an edge vector field are given as follows:

(1) $$\vec{n}=\frac{1}{c}\nabla P\cdot \nabla^{2}P$$

and

(2) $$\vec{e}=-\frac{\partial P}{\partial y}\hat{i}+\frac{\partial P}{\partial x}\hat{j},$$

where *c* is a normalization factor, and $\hat{i}$, $\hat{j}$ are unit vectors in *x* and *y* directions, respectively.

**Figure 1:**
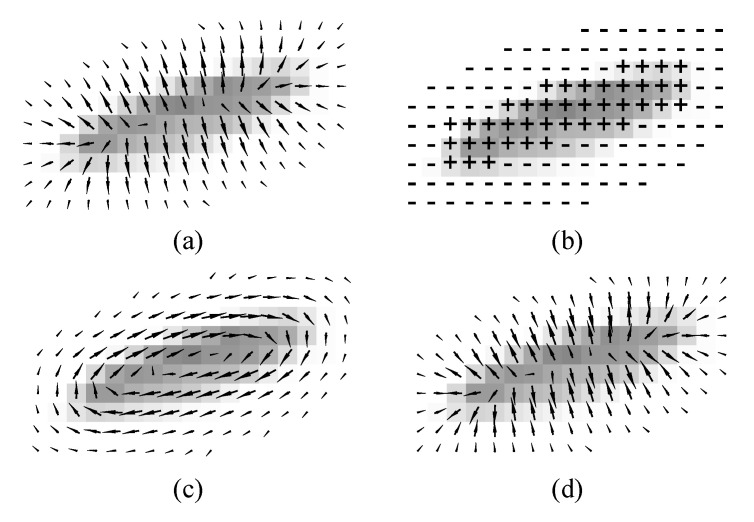
(**a**) ∇P, (**b**) $\nabla^{2}P$, (**c**) an edge vector field $\vec{e}$, and (**d**) a normal compressive vector field $\vec{n}$.

In general, in Equations (1) and (2), partial derivatives can be approximated using difference operators such as Sobel operators. Figure 1 illustrates examples of the gradient ∇P, Laplacian $\nabla^{2}P$, edge vector field $\vec{e}$, and the gradient–Laplacian vector field $\vec{n}$. In order to extract object boundaries, sequences of boundary points were obtained from trajectories of a particle driven by the combined force field $\alpha \vec{e}+\beta \vec{n}$, computed as follows:

(3) $${\vec{P}}_{k+1}={\vec{P}}_{k}+\alpha {\vec{e}}_{k}+\beta {\vec{n}}_{k},$$

where ${\vec{P}}_{k}$ is the kth particle position vector; ${\vec{e}}_{k}$ is the edge vector, interpolated at the kth particle position; ${\vec{n}}_{k}$ is the normal compressive vector, interpolated at the kth particle position; α is a tangential stepping factor, with α>0 for a particle moving in a clockwise direction and α<0 for a particle moving in a counter-clockwise direction; and β, β>0 is a normal stepping factor allowing the trajectory to converge to a boundary line.

**Figure 2:**
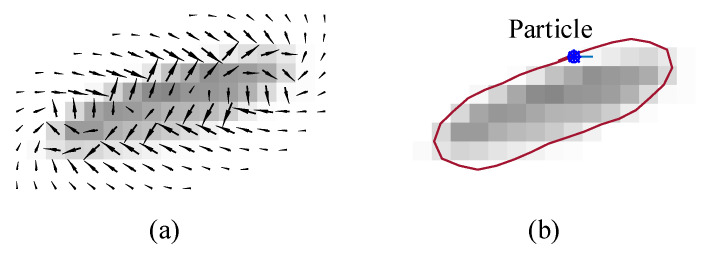
(**a**) a combined vector field, $\alpha \vec{e}+\beta \vec{n}$, α=0.5, β=0.5, and (**b**) a boundary extraction result.

Figure 2 demonstrates a combined vector field, $\alpha \vec{e}+\beta \vec{n}$, α=0.5, β=0.5, and a boundary extraction result obtained from a particle trajectory, according to Equation (3), as applied to the image in Figure 1. The PMVIF works well in extracting boundaries of regions with a constant intensity in grayscale images, providing subpixel resolution results. Nevertheless, the limitation of the PMVIF method is that the edge and normal compressive vector fields are derived from partial derivative operations that can only be applied to a scalar or intensity image. In the case of color or multispectral images in which each pixel is considered as a vector, there is no exact definition of gradient and Laplacian operators, limiting the application of the PMVIF method to color images. To overcome this limitation, a new scheme to generate a normal compressive vector field and an edge vector field for the vector image is required.

## 3. Methodology

The PMVIF algorithm requires both normal compressive and edge vector fields as particle driving forces. Due to the gradient definition that is applied only to a scalar image, the original PMVIF method can be applied only to intensity images. In this paper, the PMLCD method for finding the normal compressive and edge vector fields for color images using the center to centroid vectors of local color distance images is presented below.

### 3.1. Image Moments

For a discrete image I(x,y), a two-dimensional moment of order (p,q) [36] is defined as

(4) $$M_{pq}=\sum_{x}\sum_{y}x^{p}y^{q}I\left(x,y\right)$$

Analogous to a center of gravity in classical mechanics, the centroid $\left(\bar{x},\bar{y}\right)$ of an image I(x,y) can be calculated as follows:

(5) $$\begin{array}{cc} \left(\bar{x},\bar{y}\right) & =\left(\frac{M_{10}}{M_{00}},\frac{M_{01}}{M_{00}}\right)\\ \; & =\left(\frac{\sum_{x}\sum_{y}xI\left(x,y\right)}{\sum_{x}\sum_{y}I\left(x,y\right)},\frac{\sum_{x}\sum_{y}yI\left(x,y\right)}{\sum_{x}\sum_{y}I\left(x,y\right)}\right) \end{array}$$

The displacement between a center and a centroid of an image indicates an unbalanced pixel intensity distribution in a spatial domain.

### 3.2. Local Color Distance Images

In general, image segmentation can be viewed as a process to determine in which region each pixel should be located. For a multispectral image *I*, one feature that is widely used to determine whether or not pixels should belong to the same region is the color distance between two pixels, defined as

(6) $$D_{c}\left((x,y),(i,j)\right)=\sqrt{{\left(I_{1}\left(x,y\right)-I_{1}\left(i,j\right)\right)}^{2}+{\left(I_{2}\left(x,y\right)-I_{2}\left(i,j\right)\right)}^{2}+\ldots +{\left(I_{n}\left(x,y\right)-I_{n}\left(i,j\right)\right)}^{2}}$$

where $I_{n}\left(x,y\right)$ and $I_{n}\left(i,j\right)$ are the *n*th color components of pixels (x,y) and (i,j), respectively. In the data classification aspect, the color distance functions as a dissimilarity measurement between two pixels. Using the concept of a moving window, a local color distance image (LCD) of the pixels surrounding pixel (i,j) can be computed as

(7) $$LCD\left(x-i,y-j\right)=\underset{(x,y)\in N(i,j)}{D_{c}\left((x,y),(i,j)\right)}$$

where N(i,j) is a neighbor area of a center pixel (i,j).

**Figure 3:**
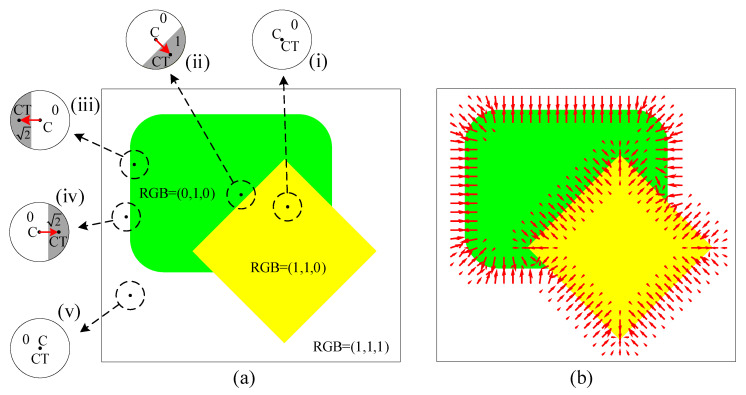
(**a**) local color distance images and (**b**) a normal compressive vector field obtained from (*C*-*to*-*CT*) vectors.

Each pixel in LCD(x−i,y−j) represents a color distance between a neighboring pixel (x,y) and the center pixel (i,j). Figure 3a illustrates examples of RGB local color distance images (i)–(v), obtained using a circular moving window computed at various places in a simple two-object image. As seen in the (i) and (v) cases, if a circular window is placed entirely inside one region, a local color distance image contains all zero pixels. Conversely, if a circular window is located at the border between two regions, the obtained local color distance image comprises pixels, with large values packed to one side of the image, as shown in cases (ii)–(iv) in Figure 3a. As a result, the centroid CT of the local color distance image computed using Equation (5) is shifted from the center *C* toward a high color distance area belonging to an adjacent region. Thus, for a local color distance image located in the proximity of a boundary, a vector from center *C* to a centroid CT points in the direction of the nearest boundary, independent of the side of the center of the local color distance on which the image is; for example, cases (iii) and (iv) in Figure 3a.

### 3.3. The Normal Compressive Vector Field

By gathering (*C*-*to*-*CT*) vectors of local color distance images obtained at all valid positions in an original image, a normal compressive vector field $\vec{n}$ can be computed as

(8) $$\vec{n}\left(i,j\right)=\frac{1}{C}\left[\begin{array}{c} {\bar{x}}_{\left(i,j\right)}-i\\ {\bar{y}}_{\left(i,j\right)}-j \end{array}\right]$$

where *C* is a normalization factor making $max\left|\vec{n}\left(i,j\right)\right|=1$, and $\left({\bar{x}}_{\left(i,j\right)},{\bar{y}}_{\left(i,j\right)}\right)$ is a centroid, computed using Equation (5), of LCD(x−i,y−j) computed using Equation (7). Figure 3b demonstrates the $\vec{n}$ of the image in Figure 3a. By combining Equations (5)–(7), $\vec{n}\left(i,j\right)$ can be directly computed as

(9) $$\vec{n}\left(i,j\right)=\frac{1}{C}\left[\begin{array}{c} \sum_{\left(x,y)\in N(i,j\right)}\left(x-i\right)D_{c}\left((x,y),(i,j)\right)/\sum_{\left(x,y)\in N(i,j\right)}D_{c}\left((x,y),(i,j)\right)\\ \sum_{\left(x,y)\in N(i,j\right)}\left(y-j\right)D_{c}\left((x,y),(i,j)\right)/\sum_{\left(x,y)\in N(i,j\right)}D_{c}\left((x,y),(i,j)\right) \end{array}\right]$$

**Figure 4:**
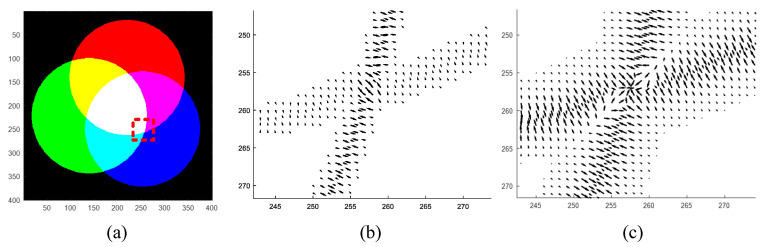
(**a**) original color image, (**b**) $\vec{n}$ obtained from Equation (1), and (**c**) $\vec{n}$ obtained from Equation (9).

It is worth noting that, in this vector field, the phenomenon that a vector on one side always points in the opposite direction to a vector on another side is called the normal compressive property. In the PMVIF technique, the normal compressive property of the vector field causes a particle to cling to the object boundary. The difference in the $\vec{n}$ from Equations (1) and (9) is that the vector size obtained from Equation (1) is smaller than Equation (9), as shown in Figure 4. As Equation (9) uses the principle of LCD while Equation (1) only uses grayscale images that include intensity for each band collapsed together and using a gradient, resulting in a smaller vector size, Equation (9) is more suitable when used with color images.

### 3.4. The Edge Vector Field

The edge vector field in the original PMVIF method, used to drive a particle to move in a direction parallel to object edges in a grayscale image, is derived from a Hamiltonian gradient vector field. However, such a vector field cannot be generated in the case of vector images such as color images where each pixel is represented by a color vector. In order to create a vector field analogous to the edge vector field, firstly, a vector-to-scalar conversion scheme must be applied to a color image to achieve a unique condition, ensuring that different colors, normally represented by vectors, are represented by different scalar values. The linearization technique used to convert a color image into a scalar auxiliary image, based on the number base system, is proposed in this paper as follows:

(10) $$Aux\left(x,y\right)=m^{\left(n-1\right)}I_{n}\left(x,y\right)+m^{\left(n-2\right)}I_{n-1}\left(x,y\right)+\ldots +m^{2}I_{3}\left(x,y\right)+mI_{2}\left(x,y\right)+I_{1}\left(x,y\right)$$

where *m* is the maximum intensity level of each color component. The auxiliary image is created to determine whether a neighbor pixel (x,y) has the same color as the center pixel (i,j).

Thus, only a difference between Aux(x,y) and Aux(i,j) is sufficient to determine whether both pixels (x,y) and (i,j) have the same color.

To obtain a gradient-like vector field, in a normal compressive vector field, as demonstrated in Figure 3b, vectors outside objects must be reverted while vectors inside objects retain the same direction. Thus, Equation (9) is modified by multiplying the local color distance with the sign of a difference between auxiliary image pixels as follows:

(11) $$\begin{array}{cc} \vec{G}\left(i,j\right) & =\left[\begin{array}{c} G_{x}\\ G_{y} \end{array}\right]\\ \; & =\frac{1}{C}\left[\begin{array}{c} \sum_{\left(x,y)\in N(i,j\right)}\left(x-i\right)sign\left(Aux\left(x,y\right)-Aux\left(i,j\right)\right)D_{c}\left(\left(x,y\right),\left(i,j\right)\right)\\ \sum_{\left(x,y)\in N(i,j\right)}\left(y-i\right)sign\left(Aux\left(x,y\right)-Aux\left(i,j\right)\right)D_{c}\left(\left(x,y\right),\left(i,j\right)\right) \end{array}\right] \end{array}$$

where *C* is a normalization factor so that $max\left|\vec{G}\left(i,j\right)\right|=1$ and $sign\left(A\right)=\left\{\begin{array}{cc} 1 & A\geq 0\\ -1 & A<0 \end{array}\right.$.

**Figure 5:**
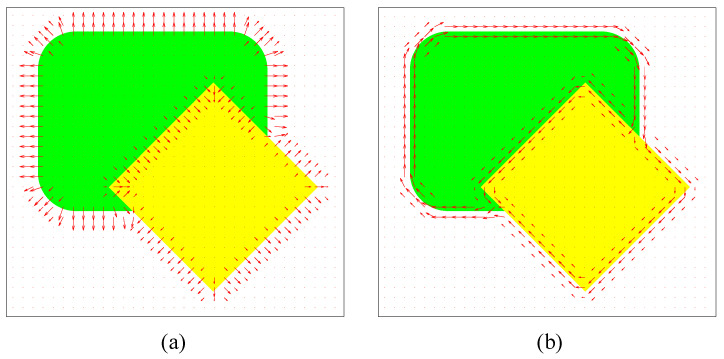
(**a**) a gradient-like vector field $\vec{G}$ obtained from Equation (11), and (**b**) an edge vector field $\vec{e}$ obtained from Equation (12).

As a result, the normal compressive property of $\vec{n}$ in Equation (9)—i.e., a vector on one side always points in a direction opposite to a vector on another side, as shown in Figure 3b—is transformed to a gradient-like property of $\vec{G}$ where vectors on both sides of objects always point in the same direction, as shown in Figure 5a. Next, by rotating all vectors in $\vec{G}$ by 90${}^{\circ }$, an edge vector field, similar to a Hamiltonian gradient vector field, is obtained as

(12) $$\vec{e}\left(i,j\right)=\left[\begin{array}{c} -G_{y}\\ G_{x} \end{array}\right]$$

as shown in Figure 5b. Notice that vectors in $\vec{e}$ in the proximity of boundaries are always larger than areas farther away. Therefore, the magnitude of $\vec{e}$ can be used as a measurement for localizing object edges.

### 3.5. Particle Motion in a Vector Image Field Derived from Local Color Distance Images

The proposed boundary extraction algorithm is based on particle motion in a vector image field derived from local color distance images (PMLCD), using a normal compressive vector field that is calculated using Equation (9) while the edge vector field is calculated using Equation (12) so that particle trajectories, calculated using Equation (3), can be obtained. The object boundaries can then be extracted from a collection of these trajectories. The remaining steps of the PMLCD method are the same as for those of the PMVIF method.

### 3.6. Appropriate PMLCD Parameter Setting

The PMLCD method has three parameters: $T_{|\vec{e}|}$, α, and β. The $T_{|\vec{e}|}$ parameter sets the threshold of $|\vec{e}|$ to determine the starting points of the particle, while α is the strength of the particle moving in the direction parallel to the object edges, and β is the strength in which the particle attaches to the object edges. These parameters are difficult to adjust. This article, therefore, suggests methods for adjusting all three parameters as follows:

$T_{|\vec{e}|}$ is determined using the Otsu’s threshold method [14]. α and β are related as follows:

(13) $$\alpha =\frac{1}{2}+R$$

(14) $$\beta =\frac{1}{2}-R$$

(15) $$R=|\left(\bar{V_{c}}V_{\vec{e}}\right)-V_{\vec{n}}|*\;100\gamma$$

where

$\bar{V_{c}}$ is the mean of the normalized variance of the color image.

$V_{\vec{e}}$ is the normalized variance of $|\vec{e}|$.

$V_{\vec{n}}$ is the normalized variance of $|\vec{n}|$.

γ is the ratio of α and β, $\left\{\begin{array}{cc} 0<\gamma <1 & \alpha >\beta \\ \gamma =0 & \alpha =\beta \\ -1<\gamma <0 & \alpha <\beta \end{array}\right.$.

The variance (*V*) of each channel, converted to a vector *A*, is made up of scalar observation defined as

(16) $$V=\frac{1}{N-1}\sum_{i=1}^{N}|A_{i}-\bar{A}{|}^{2}$$

### 3.7. Overall Boundary Extraction Method

**Figure 6:**
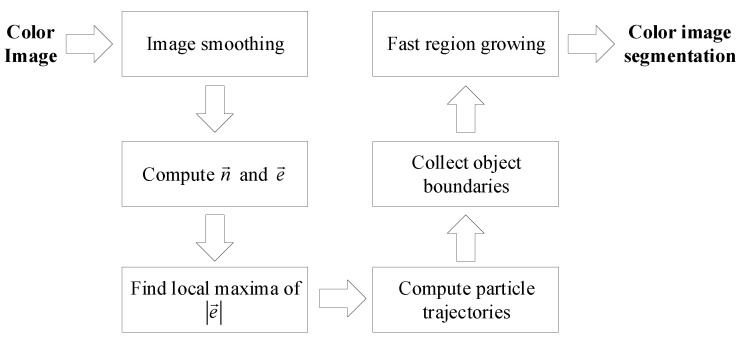
Block diagram of the proposed boundary extraction process.

The overall segmentation algorithm for color images, as illustrated in Figure 6, is described here. First, an image is smoothed to remove noise using a Gaussian low pass filter. The normal compressive vector field $\vec{n}$ and edge vector field $\vec{e}$ are then calculated using Equations (9) and (12), respectively, using a circular moving window of radius *R*. Local maximum points of $|\vec{e}|$ that are greater than a threshold are chosen as candidates for the starting points of the boundary extraction process. The suitable threshold value is determined by Otsu’s threshold method of $|\vec{e}|$. Commencing at each starting point, under the influence of a compressing edge vector field, a particle is forced to move along object edges, according to Equation (3), in both clockwise (α>0) and counter-clockwise directions (α<0) with a subpixel step size until it reaches a starting point or other previously extracted paths. Consequently, boundaries are collected from all obtained particle trajectories. Consequently, a complete edge map is achieved by quantizing the extracted boundaries. Finally, these boundaries are labeled with the fast region growing algorithm to produce the color image segmentation result.

**Figure 7:**
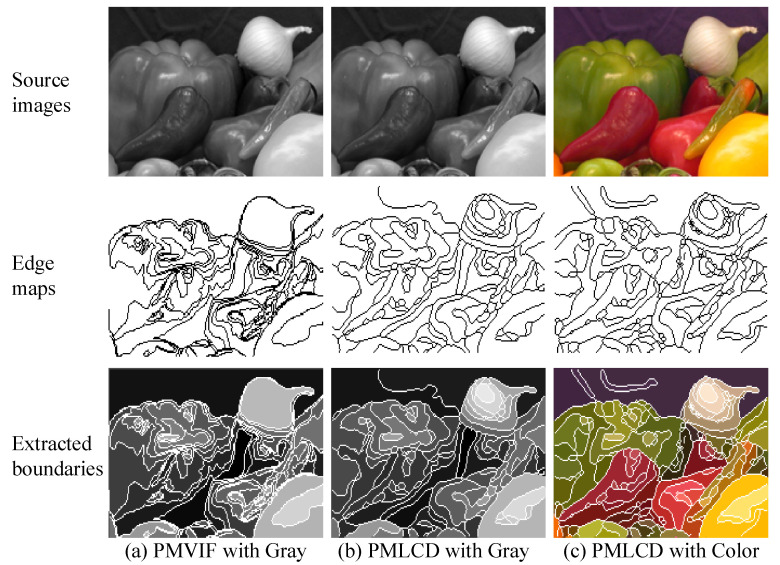
Image segmentation results obtained using (**a**) PMVIF and (**b**) PMLCD are evaluated using the same grayscale image and (**c**) the PMLCD result evaluated using the original color image.

Figure 7 illustrates image segmentation results obtained using both PMVIF and PMLCD evaluated using the same grayscale image and the original color image. Parameters used in all cases were $T_{|\vec{e}|}$ = 0.08, α = 0.5, β = 0.2 (for both PMVIF and PMLCD), and the radius of LCD = 1 (for PMLCD). As seen in Figure 7b,c, PMLCD can be applied to both grayscale and color images. In addition, when compared to the results evaluated using the grayscale image, PMLCD evaluated using the original color image provided the best results with least fault contours.

**Figure 8:**
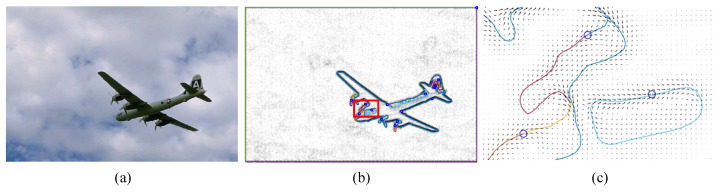
(**a**) BSDS500 #3096, (**b**) particle trajectory obtained from Equation (3), and (**c**) zoom of (**b**).

Figure 8 shows the simulation of particle motion in a vector image field derived from Equation (3) using the following parameters: radius of LCD = 1, $T_{|\vec{e}|}$ = 0.25, γ = 0.05 (α = 0.55, β = 0.45).

## 4. Experimental Results and Discussion

**Figure 9:**
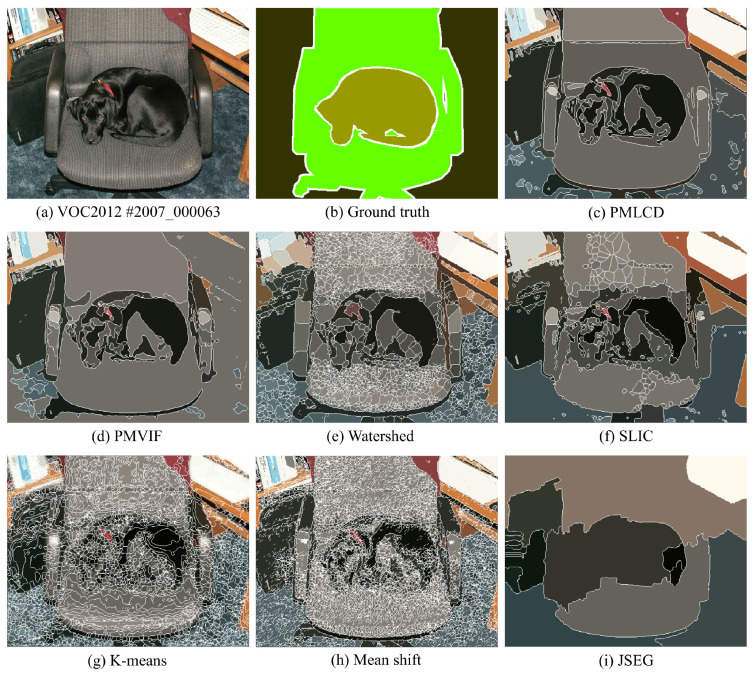
Color image segmentation results of the image #2007_000063 from VOC2012.

**Figure 10:**
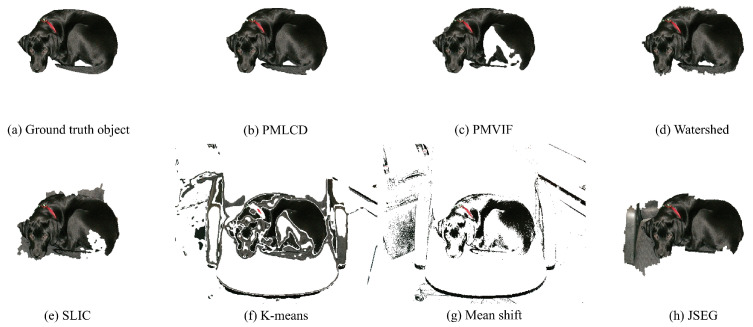
The ground truth object in Figure 9 and the corresponding segmented regions.

**Figure 11:**
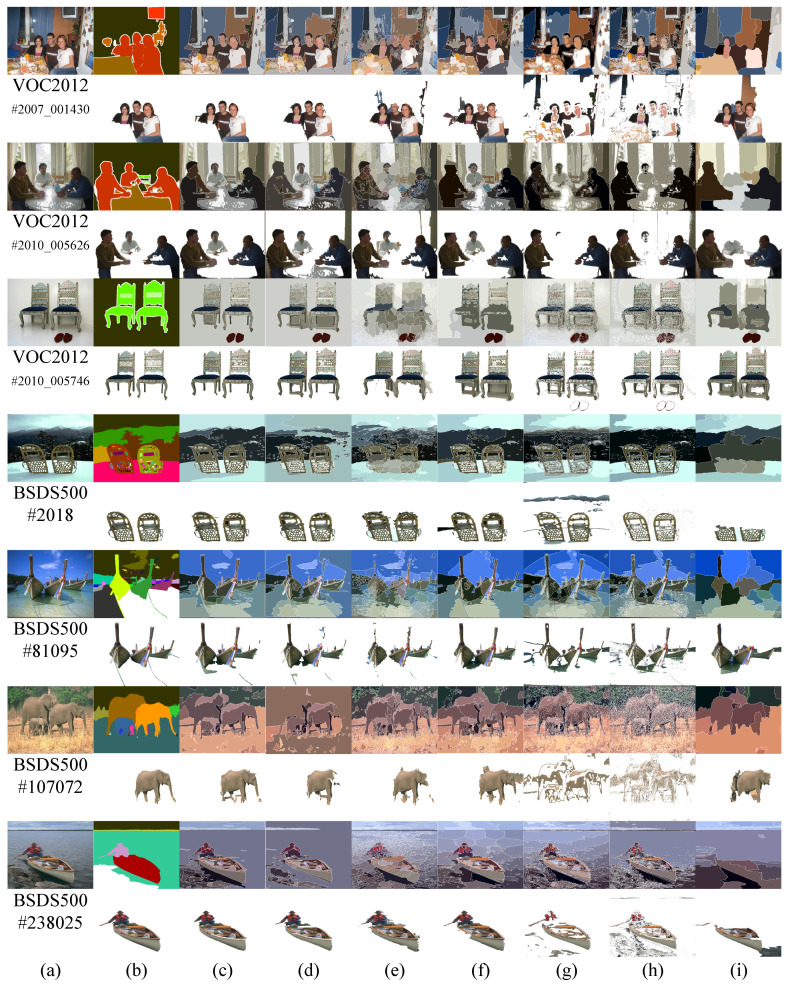
Color image segmentation results. (**a**) dataset images, (**b**) ground truths; results of (**c**) PMLCD, (**d**) PMVIF, (**e**) watershed, (**f**) SLIC, (**g**) K-means, (**h**) mean shift, and (**i**) JSEG.

**Figure 12:**
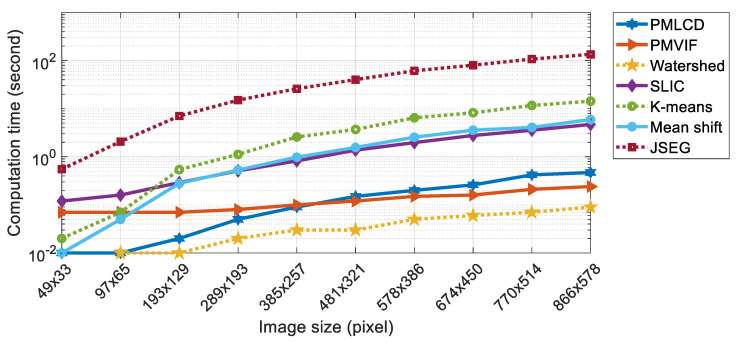
The comparison of the computation time of PMLCD and other methods.

**Figure 13:**
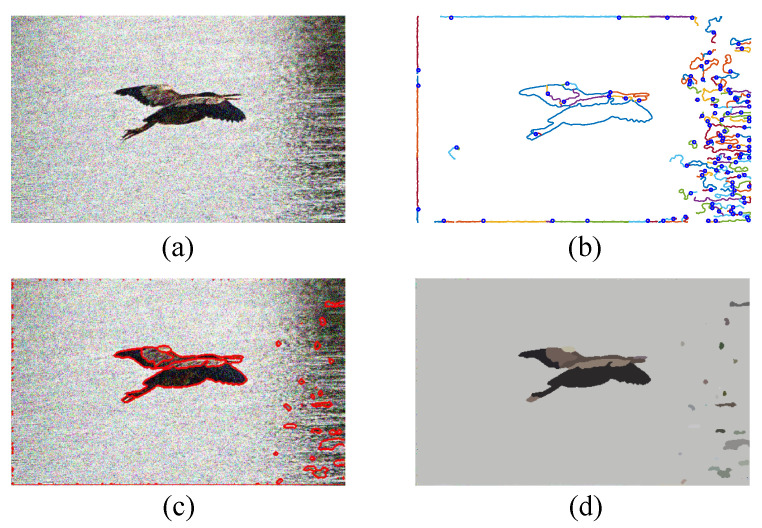
Noisy image segmentation results: (**a**) VOC2012 #2007_001289 image with SNR = 0 dB ($\sigma_{noise}$ = 0.21), (**b**) particle trajectories obtained using PMLCD with a radius of LCD = 3, $T_{|\vec{e}|}$ = 0.27, γ = −0.14 (α = 0.34, β = 0.66), (**c**) extracted boundaries (red lines), and (**d**) segmented regions.

**Figure 14:**
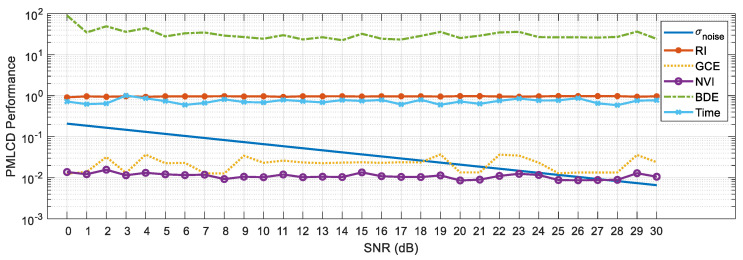
The benchmarks of the PMLCD method applied to the noisy VOC2012 #2007_001289 image.

The experimental results of color image segmentation using MATLAB 2019b with a CPU Intel Core i7-4710HQ, the VOC2012 dataset [37] and the BSDS500 dataset [38] collected to measure the performance of PMLCD compared with other unsupervised machine learning methods including K-means [2,3], mean shift [4,5], and JSEG [6,7] and classical methods including the grayscale PMVIF [21,22,23], grayscale watershed [26,27], and SLIC [34,35] are given in this section. Benchmarks used in this paper include the Rand Index (RI) [39], Global Consistency Error (GCE) [39], Normalized Variation of information (NVI) [40], Boundary Displacement Error (BDE) [39], Dice coefficient [39], computation time, and noise tolerance. Figure 9 shows the experimental color image segmentation results obtained from all methods using the image #2007_000063 from VOC2012. Figure 10 shows similarities between the object chosen from the ground truth image (a dog) and the corresponding segmented regions obtained from all methods in Figure 9. Figure 11 shows the results of the same experiment as those of Figure 9 and Figure 10 for the images randomly selected from VOC2012 and BSDS500. As shown in Table 1, the parameters of all methods for each image in the experiment have been adjusted to achieve high RI and high Dice coefficients. The average benchmarking results show that the method with the highest average RI is PMLCD, at 0.78 (0.11). The methods with the lowest average GCE are PMLCD, at 0.13 (0.05), and Watershed, at 0.13 (0.08). The methods with the lowest average NVI are JSEG, at 0.12 (0.01), and PMLCD, at 0.12 (0.04). The method with the lowest average BDE is SLIC, at 11.82 (4.12). The method with the fastest average calculation time is Watershed, at 0.06 (0.01) seconds. The method with the highest average Dice coefficient is PMLCD, at 0.93 (0.03). Briefly, the PMLCD method yields the four best average values for the RI, GCE, NVI, and Dice coefficient. Figure 12 shows the graphs of computation times used to segment image #3096 from the BSDS500, interpolated to achieve various image sizes. As seen, the Watershed, PMVIF, and PMLCD methods are the fastest, respectively, but the PMLCD is the only true color image segmentation method. Figure 13 demonstrates the result of the noisy color image segmentation of the image #2007_001289 from VOC2012 with additive white Gaussian noise (signal-to-noise ratio (SNR) 0 dB ($\sigma_{noise}$ = 0.21)) obtained using the PMLCD algorithm with the following parameters: radius of LCD = 3, $T_{|\vec{e}|}$ = 0.27 derived from Otsu’s method, γ = −0.14 resulting in α = 0.34 and β = 0.66 obtained from Equations (13) and (14), respectively. The result gives the following benchmarks: RI = 0.91, GCE = 0.01, NVI = 0.01, BDE = 90.23 and computation time = 0.72 s. Figure 14 shows the SNR-performance graph of the PMLCD applied to this image, reflecting the high noise tolerance of the PMLCD method.

## 5. Conclusions

The PMLCD color image segmentation algorithm is developed from the traditional method of particle motion in a vector image field (PMVIF) which uses two vector fields orthogonal to each other—namely a normal compressive vector field and an edge vector field—to force a particle to travel along object boundaries. Unlike the formulae previously used in the original PMVIF method, a normal compressive vector field is derived from the center-to-centroid vectors of local color distant images, whereas a gradient-like vector field is derived from center-to-centroid vectors of local color distant images in which each pixel is multiplied by the difference of the auxiliary pixels. An edge vector is then obtained by rotating each vector in a gradient-like vector field by 90${}^{\circ }$ to achieve a Hamiltonian gradient-like field. In addition, for ease of use, a method for adjusting parameters related to particle movement, including $T_{|\vec{e}|}$, α, and β, is introduced. Experimental results show that the proposed method yields promising results, with better RI, GCE, NVI, and Dice measures as well as a faster computation time and good noise resistance. Since the proposed algorithm is based on color distance measurement, which can be applied to both scalar and vector images, it outperforms other grayscale-based methods, especially in regions in which edge information cannot be visualized in the grayscale image domain. Moreover, the method is not only useful for segmenting color images, but also can be used for all types of color space and vector images including multispectral and hyperspectral images.

## Figures and Tables

**Table 1:** The performance of each method from Figure 9, Figure 10 and Figure 11.

Dataset	Method	By Image					By Object	Parameter
		RI	GCE	NVI	BDE	Time	Dice	
VOC2012 #2007_000063	PMLCD	**0.67**	0.09	0.15	16.66	0.21s	**0.97**	LCD radius 1, $T_{|\vec{e}|}$0.17(Otsu), γ0.06(α0.52,β0.48)
	PMVIF	0.63	0.30	0.15	14.87	0.49s	0.87	$T_{|\vec{e}|}$0.16(Otsu), γ0.06(α0.50,β0.50)
	Watershed	0.62	**0.06**	0.34	13.96	**0.08s**	0.96	Level 0.10
	SLIC	0.64	0.15	0.20	**13.87**	12.99s	0.88	Number of SuperPixels 20
	K-means	0.63	0.23	0.24	14.02	12.78s	0.63	Number of clusters 100
	Mean shift	0.62	0.29	0.28	13.92	147.71s	0.47	Bandwidth 0.02
	JSEG	0.66	0.30	**0.12**	18.69	84.38s	0.79	Color quantization 20
VOC2012 #2007_001430	PMLCD	**0.62**	0.09	0.17	18.75	0.41s	**0.95**	LCD radius 2, $T_{|\vec{e}|}$0.22(Otsu), γ0.00(α0.50,β0.50)
	PMVIF	0.60	0.14	0.20	16.59	0.75s	0.90	$T_{|\vec{e}|}$0.13(Otsu), γ0.00(α0.50,β0.50)
	Watershed	0.60	**0.08**	0.23	16.85	**0.07s**	0.90	Level 0.08
	SLIC	0.60	0.11	0.18	**16.05**	3.92s	0.90	Number of SuperPixels 30
	K-means	0.58	0.49	0.16	16.98	2.12s	0.41	Number of clusters 8
	Mean shift	0.57	0.47	0.18	18.11	53.50s	0.43	Bandwidth 0.07
	JSEG	**0.62**	0.16	**0.13**	21.92	60.72s	0.83	Color quantization 10
VOC2012 #2010_005626	PMLCD	**0.65**	**0.12**	0.17	17.43	0.39s	**0.90**	LCD radius 2, $T_{|\vec{e}|}$0.16(Otsu), γ0.30(α0.70,β0.30)
	PMVIF	0.60	0.24	0.20	16.24	0.64s	0.78	$T_{|\vec{e}|}$0.12(Otsu), γ0.30(α0.50,β0.50)
	Watershed	0.60	0.17	0.27	19.33	**0.07s**	0.83	Level 0.05
	SLIC	0.64	0.13	0.17	**15.54**	4.35s	0.88	Number of SuperPixels 20
	K-means	0.63	0.40	0.14	16.73	2.03s	0.78	Number of clusters 8
	Mean shift	**0.65**	0.38	**0.09**	19.75	1.96s	0.79	Bandwidth 0.25
	JSEG	0.64	0.20	0.14	20.19	71.54s	0.85	Color quantization 19
VOC2012 #2010_005746	PMLCD	**0.82**	**0.05**	**0.09**	7.65	0.19s	**0.93**	LCD radius 1, $T_{|\vec{e}|}$0.21(Otsu), γ0.00(α0.50,β0.50)
	PMVIF	0.73	**0.05**	0.12	**4.26**	0.45s	0.91	$T_{|\vec{e}|}$0.18(Otsu), γ0.00(α0.50,β0.50)
	Watershed	0.37	0.11	0.30	19.40	**0.07s**	0.82	Level 0.01
	SLIC	0.46	0.16	0.15	7.59	4.13s	0.75	Number of SuperPixels 5
	K-means	0.37	0.16	0.24	9.28	10.62s	0.74	Number of clusters 100
	Mean shift	0.41	0.17	0.23	13.43	373.74s	0.71	Bandwidth 0.02
	JSEG	0.46	0.14	0.14	14.47	36.85s	0.77	Color quantization 2
BSDS500 #2018	PMLCD	**0.90**	**0.21**	**0.09**	5.43	0.20s	**0.92**	LCD radius 1, $T_{|\vec{e}|}$0.24(Otsu), γ0.35(α0.86,β0.14)
	PMVIF	0.75	0.46	0.14	4.32	0.49s	**0.92**	$T_{|\vec{e}|}$0.19(Otsu), γ0.35(α0.52,β0.48)
	Watershed	0.84	0.31	0.16	7.88	**0.05s**	0.83	Level 0.04
	SLIC	0.89	0.22	0.10	**3.75**	3.54s	0.88	Number of SuperPixels 10
	K-means	0.82	0.61	0.16	4.14	2.33s	0.69	Number of clusters 10
	Mean shift	0.74	0.36	0.12	5.01	8.34s	0.73	Bandwidth 0.10
	JSEG	0.81	0.37	0.11	18.94	69.68s	0.59	Color quantization 30
BSDS500 #81095	PMLCD	**0.90**	0.17	**0.09**	9.55	0.35s	**0.89**	LCD radius 2, $T_{|\vec{e}|}$0.21(Otsu), γ0.12(α0.64,β0.36)
	PMVIF	0.81	0.26	0.14	9.64	0.38s	0.86	$T_{|\vec{e}|}$0.18(Otsu), γ0.12(α0.50,β0.50)
	Watershed	0.85	**0.15**	0.18	10.63	**0.06s**	0.84	Level 0.05
	SLIC	0.85	0.20	0.11	9.99	2.50s	0.86	Number of SuperPixels 10
	K-means	0.81	0.49	0.16	**8.77**	2.77s	0.64	Number of clusters 14
	Mean shift	0.82	0.50	0.14	10.41	38.64s	0.63	Bandwidth 0.08
	JSEG	0.84	0.30	0.11	16.63	47.30s	0.80	Color quantization 12
BSDS500 #107072	PMLCD	**0.85**	0.18	**0.10**	12.78	0.16s	**0.93**	LCD radius 1, $T_{|\vec{e}|}$0.22(Otsu), γ-0.05(α0.47,β0.53)
	PMVIF	0.48	0.33	0.13	15.34	0.27s	0.47	$T_{|\vec{e}|}$0.23(Otsu), γ-0.05(α0.50,β0.50)
	Watershed	0.75	**0.12**	0.25	15.50	**0.05s**	0.92	Level 0.15
	SLIC	0.76	**0.12**	0.16	12.27	3.89s	0.88	Number of SuperPixels 25
	K-means	0.75	0.34	0.17	12.33	3.61s	0.43	Number of clusters 20
	Mean shift	0.74	0.32	0.27	13.58	79.77s	0.41	Bandwidth 0.02
	JSEG	0.82	0.22	**0.10**	**8.99**	47.27s	0.73	Color quantization 10
BSDS500 #238025	PMLCD	**0.86**	0.10	**0.09**	13.60	0.37s	**0.96**	LCD radius 2, $T_{|\vec{e}|}$0.19(Otsu), γ0.15(α0.64,β0.36)
	PMVIF	0.69	0.31	0.10	15.95	0.28s	0.93	$T_{|\vec{e}|}$0.18(Otsu), γ0.15(α0.50,β0.50)
	Watershed	0.65	**0.06**	0.29	19.30	**0.06s**	0.94	Level 0.05
	SLIC	0.67	0.08	0.17	15.49	3.73s	0.95	Number of SuperPixels 30
	K-means	0.68	0.35	0.15	**12.80**	2.94s	0.67	Number of clusters 16
	Mean shift	0.71	0.47	0.12	13.41	50.11s	0.61	Bandwidth 0.05
	JSEG	0.73	0.24	0.11	15.27	42.22s	0.61	Color quantization 10
**Average (Standard Deviation)**	**PMLCD**	**0.78** (0.11)	**0.13** (0.05)	**0.12** (0.04)	12.73 (4.52)	0.29s (0.10)	**0.93** (0.03)	LCD radius 1.50(0.50), γ0.14(0.15)
	**PMVIF**	0.66 (0.10)	0.26 (0.12)	0.15 (0.03)	12.15 (4.98)	0.47s (0.16)	0.83 (0.14)	γ0.14(0.15)
	**Watershed**	0.66 (0.15)	**0.13** (0.08)	0.25 (0.06)	15.36 (4.03)	**0.06s** (0.01)	0.88 (0.05)	Level 0.0.07(0.04)
	**SLIC**	0.69 (0.13)	0.15 (0.04)	0.16 (0.03)	**11.82** (4.12)	4.88s (3.11)	0.87 (0.05)	Number of SuperPixels 18.75(8.93)
	**K-means**	0.66 (0.14)	0.38 (0.14)	0.18 (0.04)	11.88 (4.05)	4.90s (3.99)	0.62 (0.13)	Number of clusters 34.50(38.01)
	**Mean shift**	0.66 (0.13)	0.37 (0.10)	0.18 (0.07)	13.45 (4.21)	94.22s (113.90)	0.60 (0.14)	Bandwidth 0.08(0.07)
	**JSEG**	0.70 (0.12)	0.24 (0.07)	**0.12** (0.01)	16.89 (3.78)	57.50s (15.60)	0.75 (0.09)	Color quantization 14.13(8.01)
